# LncRNA HOXA-AS2 promotes the progression of prostate cancer via targeting miR-509-3p/PBX3 axis

**DOI:** 10.1042/BSR20193287

**Published:** 2020-08-13

**Authors:** Shangwen Xiao, Bin Song

**Affiliations:** 1 Department of Urology, Ankang Traditional Chinese Medicine Hospital, Ankang 725000, Shaanxi, China; 2 Department of Urology, Tangdu Hospital, Air Force Medical University, No. 569 Xinsi Road, Baqiao District, Xi’an 710038, Shaanxi, China

**Keywords:** competing endogenous RNA (ceRNA), HOXA-AS2, miR-509-3p, PBX3, prostate cancer

## Abstract

Long non-coding RNAs (lncRNAs) act as crucial modulators during the development of diverse cancers. Although various types of lncRNAs in prostate cancer (PCa) have been explored, quantities of lncRNAs still wait to be exploited. The present study is to probe the functions and mechanism of lncRNA HOXA cluster antisense RNA 2 (HOXA-AS2) in PCa. In the present study, we discovered that HOXA-AS2 was highly expressed in PCa tissues and cells. HOXA-AS2 depletion obviously influenced cell proliferation, migration, invasion, as well as epithelial-to-mesenchymal transition (EMT) progression. In addition, miR-509-3p had low expression in PCa cells and inversely modulated by HOXA-AS2. Cutting down HOXA-AS2 expression was capable of up-regulating miR-509-3p expression. In addition, HOXA-AS2 served as a competing endogenous RNA (ceRNA) through sponging miR-509-3p to release pre-B-cell leukemia homeobox 3 (PBX3) expression. The expression of PBX3 was noticeably high in tumor tissues. And PBX3 expression level was down-regulated markedly with the knockdown of HOXA-AS2. Rescue experiments certified the facilitated role of HOXA-AS2-miR-509-3p-PBX3 axis in regulating the progress of PCa. The present study may provide clues for exploration of novel therapeutic targets for PCa patients.

## Introduction

Prostate cancer (PCa) is one of the leading causes for cancer-related mortality among the developed countries. The abnormal growth and metastasis of prostate cells are symbols of PCa progression. PCa generally occurs in males over 50 years old [1]. PCa is the sixth cause of cancer-related deaths around the world. There were 200 thousand new cases in the U.S.A. in 2013 [2]. According to Hsu PP et al. (2008), 164690 new cases were registered in U.S.A. and 29430 men died owing to PCa [3]. Therefore, it is urgent to explore the novel effective molecular targets for the diagnosis and treatment of PCa.

Long non-coding RNAs (lncRNAs) are considered as transcripts with length over 200 nt and without protein-coding ability. Of late years, lncRNAs have obtained the widespread concern as their potential in regulation of tumorigenesis and metastasis [4]. LncRNAs have been verified to be aberrantly expressed in multiple kinds of diseases, like PCa. LncRNAs result in morbidity or maintain lesion state [5]. HOXA cluster antisense RNA 2 (HOXA-AS2) is a 1048-bp lncRNA existing entre the human genes of HOXA3 and HOXA4. HOXA-AS2 can contribute to cell survival, proliferation and invasion in leukemia [6] and gallbladder carcinoma [7]. However, the expression pattern and mechanism of lncRNA HOXA-AS2 in PCa remain to be explored. Therefore, the present study focused on the impact of HOXA-AS2 on the modulation of cellular processes in PCa.

Plenty of researches have demonstrated that lncRNAs acted as competing endogenous RNAs (ceRNAs) to compete with messenger RNAs (mRNAs) for microRNAs (miRNAs) [8]. During the miRNAs modulation on their targets, ceRNAs serve as post-transcriptional modulators. miRNAs have no protein-coding ability with 20–25 nucleotide RNA without coding ability. miRNAs play significant roles in controlling gene expression by binding to the 3′-UTR region of the downstream mRNAs [9]. It was determined that HOXA-AS2 bound to miR-509-3p in PCa by using bioinformatics analyses and mechanism investigation. Recent studies have also detected that miR-509-3p affects the biological development of different cancer cells [10]. Besides, miR-509-3p was identified as a clinically significant miRNA in ovarian cancer, due to its inhibitory effect on cell migration and multicellular spheroids [11]. This paper was designed to probe the relationship between HOXA-AS2 and miR-509-3p in PCa.

Pre-B-cell leukemia homeobox 3 (PBX3) belongs to three-amino acid-loop-extension family of homeodomain transcription factors [12]. Emerging reports showed that PBX3 is up-regulated in multiple human cancers, including glioblastoma [13], hepatocellular carcinoma [14] and PCa [15]. The present study tried to unveil the role of PBX3 in HOXA-AS2-mediated PCa progression.

## Materials and methods

### Patients and tissue samples

Sixty-eight paired samples of PCa tissues and adjacent normal tissue were offered by patients who underwent surgical excision at Ankang Traditional Chinese Medicine Hospital. Tissue samples were frozen at −80°C promptly. All these experimental procedures have been approved by the Ethics Committee. Before surgery, no patients had received chemotherapy or radiotherapy. Written informed consents were obtained from patients.

### Cell culture

Human prostatic epithelial cell (RWPE) and PCa cells (LNCaP, DU145 and PC3) were purchased from American Type Culture Collection (ATCC; Manassas, VA, U.S.A.). RWPE cells were grown in Keratinocyte Serum-Free Medium (Invitrogen, Carlsbad, CA, U.S.A.) added with human recombinant EGF (Invitrogen) and bovine pituitary extract (Invitrogen). PCa cells were grown with Dulbecco’s modified Eagle’s medium (DMEM; Thermo Fisher Scientific, Waltham, MA, U.S.A.) containing 1% penicillin/streptomycin (Thermo Fisher Scientific) and 10% fetal bovine serum (FBS; Thermo Fisher Scientific). Cells were cultured at 37°C in a humidified incubator containing 5% CO_2_.

### Cell transfection

Cell transfection was conducted for 48 h via Lipofectamine 3000 (Invitrogen). Specific shRNAs against HOXA-AS2 (sh-HOXA-AS2#1, sh-HOXA-AS2#2 and sh-HOXA-AS2#3) and their corresponding NC (sh-NC) were bought from Genechem (Shanghai, China). The pcDNA3.1 vector targeting HOXA-AS2 or PBX3 and the empty vector were also acquired from Genechem. Besides, miR-509-3p mimics and NC mimics were obtained from GenePharma (Shanghai, China). Subsequently, LNCaP or DU145 cells were severally transfected with above plasmids. Cell transfection was conducted in triplicate.

### RNA isolation and qRT-PCR

Total RNA was isolated according to the instructions of TRIzol reagent (Invitrogen) and subsequently reverse-transcribed into the first-strand cDNA via a PrimeScript RT reagent kit (Takara Bio, Shiga, Japan). qRT-PCR was carried out with SYBR Green kit (Takara Bio) on an ABI Prism 5700 Sequence Detection System (Applied Biosystems, Foster City, CA, U.S.A.). Experimental processes were implemented in-line with the manuals. GAPDH or U6 was used as the internal control and relative gene expressions were determined by the 2^−ΔΔ*C*_t_^ approach. qRT-PCR assay was conducted in triplicate.

### Colony formation assay

After being plated into six-well culture dishes, transfected LNCaP or DU145 cells were cultured for 14 days. Following that, cells were fixed for 15 min in formaldehyde (Sigma–Aldrich, Burlington, Massachusetts, U.S.A.) and subsequently stained for 5 min by Crystal Violet (Sigma–Aldrich). The number of colonies was counted with visual inspection. Colony formation assay was conducted in triplicate.

### Flow cytometry

Assessment of cell apoptosis was conducted with flow cytometry by use of the Annexin V-fluorescein isothiocyanate (FITC) apoptosis detection kit (Beyotime, Shanghai, China). Transfected LNCaP or DU145 cells were rinsed by phosphate-buffered saline (PBS; Sigma–Aldrich), after which Annexin V-FITC plus propidium iodide (Sigma–Aldrich) in the dark were added, diluting with binding buffer (Thermo Fisher Scientific), and subsequently filtered through mesh cell filters. Apoptotic rate was calculated using flow cytometry (BD, Franklin Lakes, NJ, U.S.A.). Flow cytometry apoptosis assay was conducted in triplicate.

### Transwell assay

Transwell inserts (Millipore, Bedford, MA, U.S.A.) coated with or without Matrigel (BD) were used for evaluating cell migration and invasion. Transfected LNCaP or DU145 cells in serum-free medium were put into the upper chambers, while medium containing 20% FBS was added into the lower chambers. Twenty-four hours later, cells were fixed for 20 min by 4% paraformaldehyde (PFA; Sigma–Aldrich) and stained for 15 min in 0.1% Crystal Violet dye. Thereafter, cells on the inner layer were wiped off softly with a cotton swab. Migratory or invasive cells were counted at five randomly chosen views. Transwell assay was conducted in triplicate.

### Western blot

Transfected LNCaP or DU145 cells were lysed in SDS buffer (Beyotime) so as to obtain the protein for electrophoresis, and whole protein was then transferred on to PVDF membrane (Whatman, Buckinghamshire, U.K.). Primary antibodies included antibodies against E-cadherin (1/10000; ab40772, Abcam, Cambridge, U.S.A.), N-cadherin (1/1000; ab76057, Abcam), Vimentin (1/1000; ab8978, Abcam), PBX3 (1/1000; ab56239, Abcam) and GAPDH (1/2500; ab9485, Abcam) were used to incubate the membrane. GAPDH was utilized for normalizing the protein levels. Protein expression signaling was studied by enhanced chemiluminescence (ECL) substrate (Thermo Fisher Scientific). Western blot was conducted in triplicate.

### Luciferase reporter assay

HOXA-AS2-WT/MUT or PBX3-WT/MUT was subcloned into the pmirGLO dual-luciferase vector (Promega, Madison, WI, U.S.A.) to generate pmirGLO-HOXA-AS2-WT/MUT or pmirGLO-PBX3-WT/MUT. On one hand, LNCaP or DU145 cells were co-transfected with pmirGLO-HOXA-AS2-WT/MUT and miR-509-3p mimics or NC mimics, on the other hand, LNCaP or DU145 cells were co-transfected with pmirGLO-PBX3-WT/MUT and miR-509-3p mimics or miR-509-3p mimics+pcDNA3.1/HOXA-AS2 or NC mimics. After co-transfection for 48 h, Dual Luciferase Reporter Assay System (Promega) was applied in accordance with its requirements. Luciferase reporter assay was conducted in triplicate.

### RNA immunoprecipitation assay

RNA immunoprecipitation (RIP) assay was performed on the basis of EZ-Magna RIP RNA-Binding Protein Immunoprecipitation Kit (Millipore). LNCaP or DU145 cells were lysed by complete RIP lysis buffer (Sigma–Aldrich). After that, cleared lysates were incubated with RIP buffer adding magnetic beads (Invitrogen) conjugated to human anti-Ago2 antibody (1/200; 66720-1-Ig, Proteintech, Beijing, China) or normal mouse IgG (1/200; A0408, Beyotime). Subsequently, co-precipitated RNAs were subjected to qRT-PCR. RIP assay was conducted in triplicate.

### Statistical analysis

All assays were conducted in triplicate. Data were imported into GraphPad Prism 7.0 (GraphPad Software, La Jolla, CA, U.S.A.) and shown as mean ± SD. Assessment of differences was performed by Student’s *t* test or one-way ANOVA. A *P*-value <0.05 represented statistical significance. The Kaplan–Meier method was utilized to plot survival curves which were compared by log-rank test. Gene linear relationship was examined by Pearson’s correlation analysis.

## Results

### HOXA-AS2 is notably highly expressed in PCa cells

**Figure 1:**
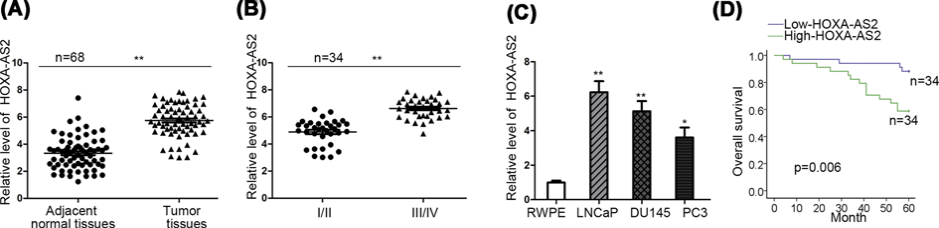
HOXA-AS2 is highly expressed in PCa cells (**A**) qRT-PCR analysis was used to detect HOXA-AS2 expression in PCa tissues and matched normal tissues. (**B**) qRT-PCR analysis was applied to assess HOXA-AS2 expression in PCa tissues in different clinical stages. (**C**) HOXA-AS2 expression in prostate epithelial cell line RWPE and PCa cells (LNCaP, DU145 and PC3) were estimated by qRT-PCR similarly. (**D**) Log-rank test was performed to evaluate patient survival rate on the basis of high or low level of HOXA-AS2. All data were displayed as the mean ± SD. *P<0.05, **P<0.01.

At first, HOXA-AS2 expression was assessed via qRT-PCR analysis. HOXA-AS2 presented higher expression level in PCa tissues in contrast with corresponding normal controls (Figure 1A). Similarly, HOXA-AS2 expression level was relatively high in patients with advanced stages than that in patients with early stages (Figure 1B). Furthermore, HOXA-AS2 expression levels were apparently strong in PCa cells (LNCaP, DU145 and PC3) in contrast with normal prostate epithelial cell RWPE (Figure 1C). LNCaP and DU145 cells were selected for the later investigations because of high HOXA-AS2 expression. Importantly, patients’ survival rate in high-HOXA-AS2 expression group was lower than those in low-HOXA-AS2 expression group (Figure 1D). These findings predicted that HOXA-AS2 served a tumor-promoting role in PCa.

### Silencing of HOXA-AS2 abates colony formation numbers and migratory ability in PCa cells

**Figure 2:**
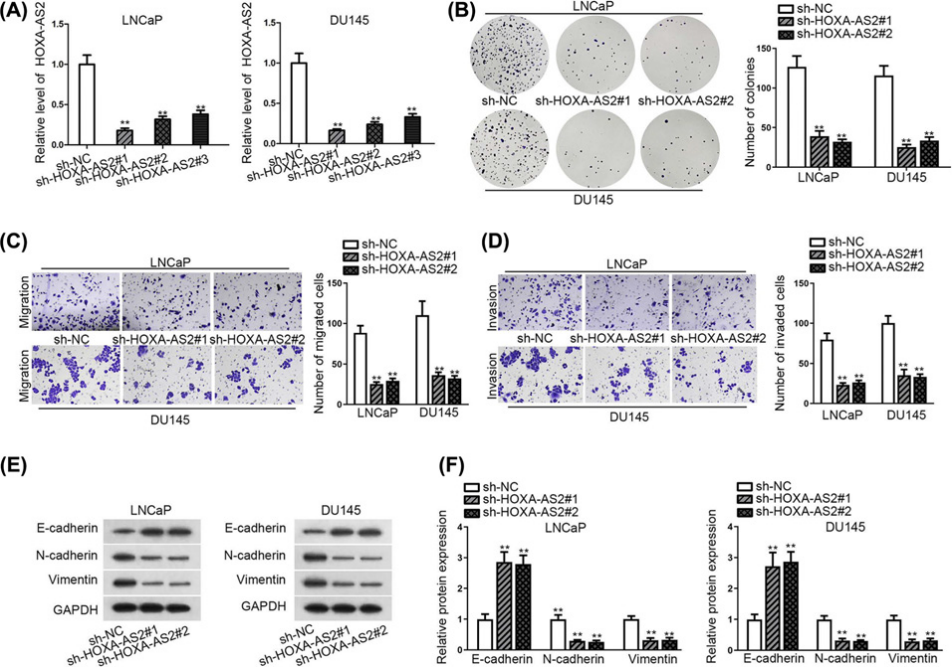
Depletion of HOXA-AS2 abates colony formation numbers and migratory ability in PCa cells (**A**) HOXA-AS2 expression was measured by qRT-PCR in selected LNCaP and DU145 cell transfected with sh-HOXA-AS2#1/#2/#3. (**B**) Colony formation assay was conducted to estimate cell proliferation after HOXA-AS2 silence. (**C,D**). Transwell assays were applied to detect the migration and invasion in HOXA-AS2-downregulated cells. (**E,F**) Western blot and qRT-PCR analyses were used to assess the expression of epithelial marker E-cadherin as well as mesenchymal markers (N-cadherin and Vimentin). All results were presented as the mean ± SD. **P<0.01.

Above all, three shRNAs targeting HOXA-AS2 were transfected into LNCaP and DU145 cells for loss-of-function study. As delineated in Figure 2A, HOXA-AS2 expression level in LNCaP and DU145 were strikingly decreased under the condition of reducing HOXA-AS. Colony formation assay demonstrated that depleted HOXA-AS2 impaired the proliferative abilities of both cancer cells (Figure 2B). The results of transwell assay demonstrated down-regulating HOXA-AS2 suppressed cell migration and invasion (Figure 2C,D). Additionally, the findings of Western blot assay and qRT-PCR analysis revealed that epithelial marker E-cadherin was up-regulated observably whereas that of the mesenchymal markers (N-cadherin and Vimentin) were declined (Figure 2E,F), hinting that the interference of HOXA-AS2 negatively affected epithelial-to-mesenchymal transition (EMT). Altogether, inhibition of HOXA-AS2 could suppress cell proliferation and migration in PCa.

### MiR-509-3p is expressed lower in PCa cells and inversely correlated with HOXA-AS2

**Figure 3:**
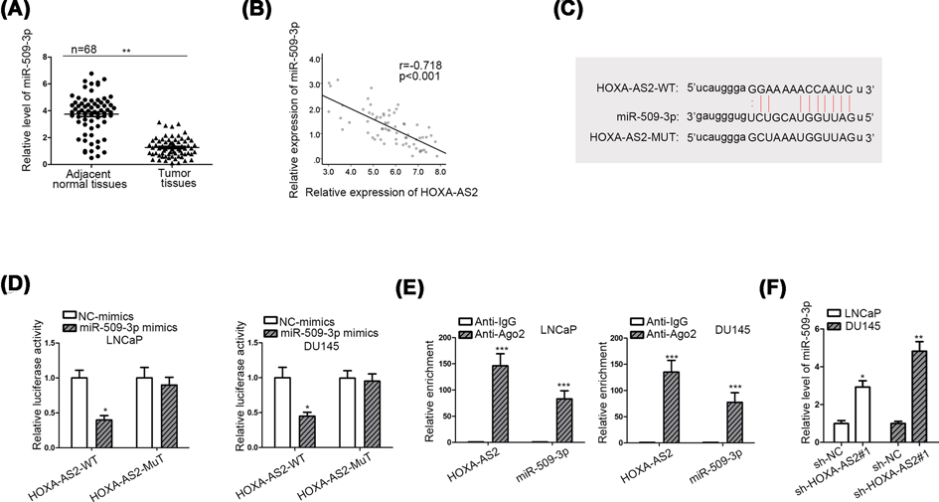
MiR-509-3p is expressed lower in PCa cells and inversely correlated with HOXA-AS2 (**A**) MiR-509-3p expression in PCa tissues and adjacent normal samples were tested by qRT-PCR. (**B**) The correlation between HOXA-AS2 and miR-509-3p in PCa tissues was examined by Pearson’s correlation analysis. (**C**) The binding sites entre HOXA-AS2 and miR-509-3p were obtained from starBase database. (**D**) The luciferase reporter assay was implemented to attest the binding relationship entre HOXA-AS2 and miR-509-3p in LNCaP and DU145 cells. (**E**) RIP assay was utilized to prove the coexistence of HOXA-AS2 and miR-509-3p in RISC. (**F**) qRT-PCR was performed to determine miR-509-3p expression in the condition of HOXA-AS2 depletion. All data were expressed as the mean ± SD. *P<0.05, **P<0.01, ***P<0.001. RISC: RNA-induced silencing complex.

Cytoplasmic lncRNAs can regulate mRNAs by sequestering miRNAs. To recognize the modulatory pattern of HOXA-AS2 in PCa, we screened on starBase website (selecting ten cancer types in pan-cancer). Through screening, we found that only miR-509-3p had the binding potential with HOXA-AS2. Besides, a number of papers have certified the cancer suppressing character of miR-509-3p in multiple cancers [16–18]. Consequently, we chose miR-509-3p as the downstream target for HOXA-AS2. The expression of miR-509-3p was first measured in paired clinical tissues. We found that miR-509-3p expression was lower in tumor tissues (Figure 3A), which was anti-correlated with HOXA-AS2 (Figure 3B). Meanwhile, we discovered the binding sites entre HOXA-AS2 and miR-509-3p (Figure 3C). Luciferase reporter assay denoted that the luciferase activity of wild-type HOXA-AS2 (HOXA-AS2-WT) was evidently cut down by co-transfection with miR-509-3p mimics (Figure 3D). RIP assay uncovered that HOXA-AS2 and miR-509-3p were both enriched in Ago2 group by contrast with that in control IgG (Figure 3E). Finally, reduction in HOXA-AS2 expression augmented miR-509-3p expression level (Figure 3F). All these data suggested the inverse relationship entre HOXA-AS2 and miR-509-3p in PCa cells.

### HOXA-AS2 functions as a ceRNA through sponging miR-509-3p to up-regulate PBX3

**Figure 4:**
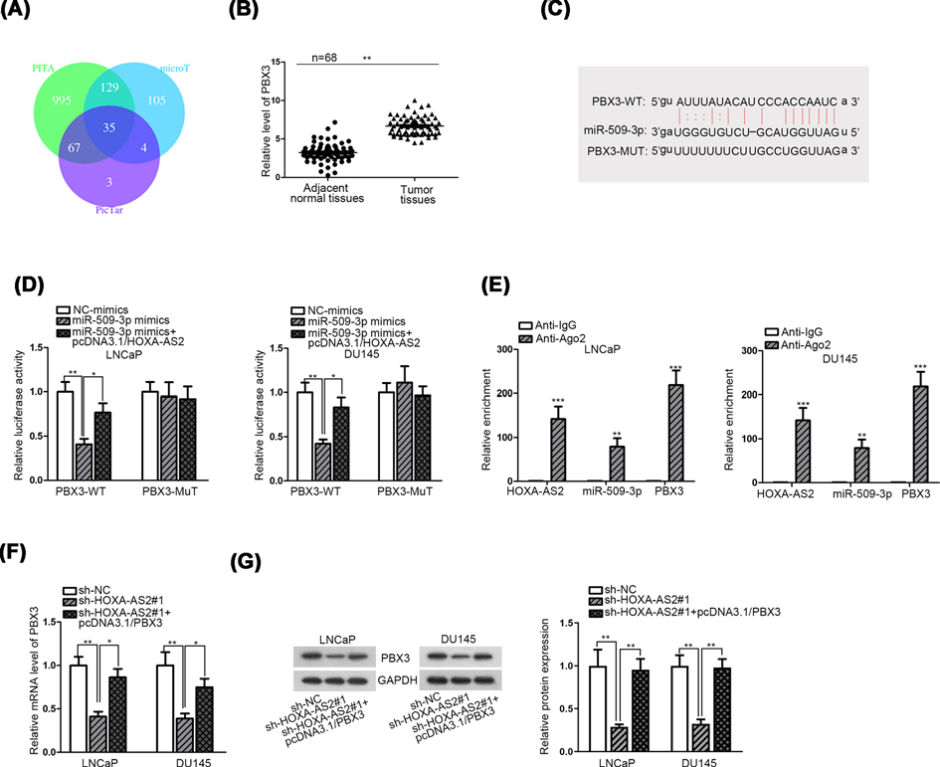
HOXA-AS2 functions as a ceRNA through sponging miR-509-3p to up-regulate PBX3 (**A**) Three websites (PITA, microT and PicTar) predicted 35 mRNAs had binding sites with miR-509-3p. (**B**) PBX3 expression in PCa tissues and adjacent normal tissues were evaluated by qRT-PCR. (**C**) The binding sites between miR-509-3p and PBX3 obtained from starBase database. (**D**) The luciferase reporter was implemented to determine the binding correlation between miR-509-3p and PBX3 in LNCaP and DU145 cells. (**E**) RIP assay was applied to assess the enrichment of HOXA-AS2, miR-509-3p and PBX3 in RISC. (**F,G**) The qRT-PCR and Western blot analyses were conducted to detect PBX3 expression of both cell lines with two interventions. All data were expressed as the mean ± SD. *P<0.05, **P<0.01, ***P<0.001.

To estimate whether HOXA-AS2 acted as a ceRNA to modulate miRNA-mRNA axis, we conducted mechanism experiments to determine the target of miR-509-3p. First, 35 target mRNAs which could bind with miR-509-3p were detected from three websites (PITA, microT and PicTar) (Figure 4A). Combination with ten cancer types in pan-cancer, four mRNAs (PBX3, KLF6, ARHGAP1, ST3GAL2) were selected out. According to the result of qRT-PCR analysis, only PBX3 was down-regulated significantly in two PCa cells transfected with sh-HOXA-AS2 (Supplementary Figure S1A). Among the candidate targets, PBX3, a member of the lately reported PBX family, has been described as a contributor to cancer cell proliferation and growth [19]. PBX3 is an mRNA which had protein-coding ability. Moreover, PBX3 has been reported as the oncogene in various human cancers [20,21], indicating its protein had the oncogenic potential. Up-regulated expression of PBX3 was associated with tumorigenesis and development in malignancies like leukemia [19,22] and gastric cancer [23,24]. Thus, PBX3 was selected as the downstream mRNA of miR-509-3p.

PBX3 expression was noticeably up-regulated in tumor tissues as depicted in Figure 4B. The possible binding sites entre miR-509-3p and 3′UTR region of PBX3 acquired from starBase online were displayed (Figure 4C). It was observed that the mRNA and protein levels of PBX3 were both decreased in LNCaP and DU145 cells after transfected with miR-509-3p mimics (Supplementary Figure S1B,C). Subsequently, luciferase reporter assay signified that augment of miR-509-3p expression induced the depletion of the luciferase activity of the wild-type PBX3 (PBX3-WT) but not the mutant type (PBX3-MUT) (Figure 4D). Subsequently, overexpressing HOXA-AS2 recovered the luciferase activity of the wild-type PBX3. The rescue assay validated that HOXA-AS2 acted as a miR-509-3p sponge. Furthermore, RIP assay exhibited that HOXA-AS2, miR-509-3p and PBX3 were enriched in Ago2-contained beads compared with negative control group (Figure 4E). Through qRT-PCR and Western blot assay, we determined the down-regulation of PBX3 in cells transfected with HOXA-AS2-specific shRNA; however, increased level of PBX3 recovered its expression impaired by HOXA-AS2 knockdown (Figure 4F,G). Taken together, HOXA-AS2 functioned as a ceRNA in PCa through serving as a sponge of miR-509-3p to modulate PBX3 expression.

### HOXA-AS2-miR-509-3p-PBX3 axis modulated PCa cell proliferation and migration

**Figure 5:**
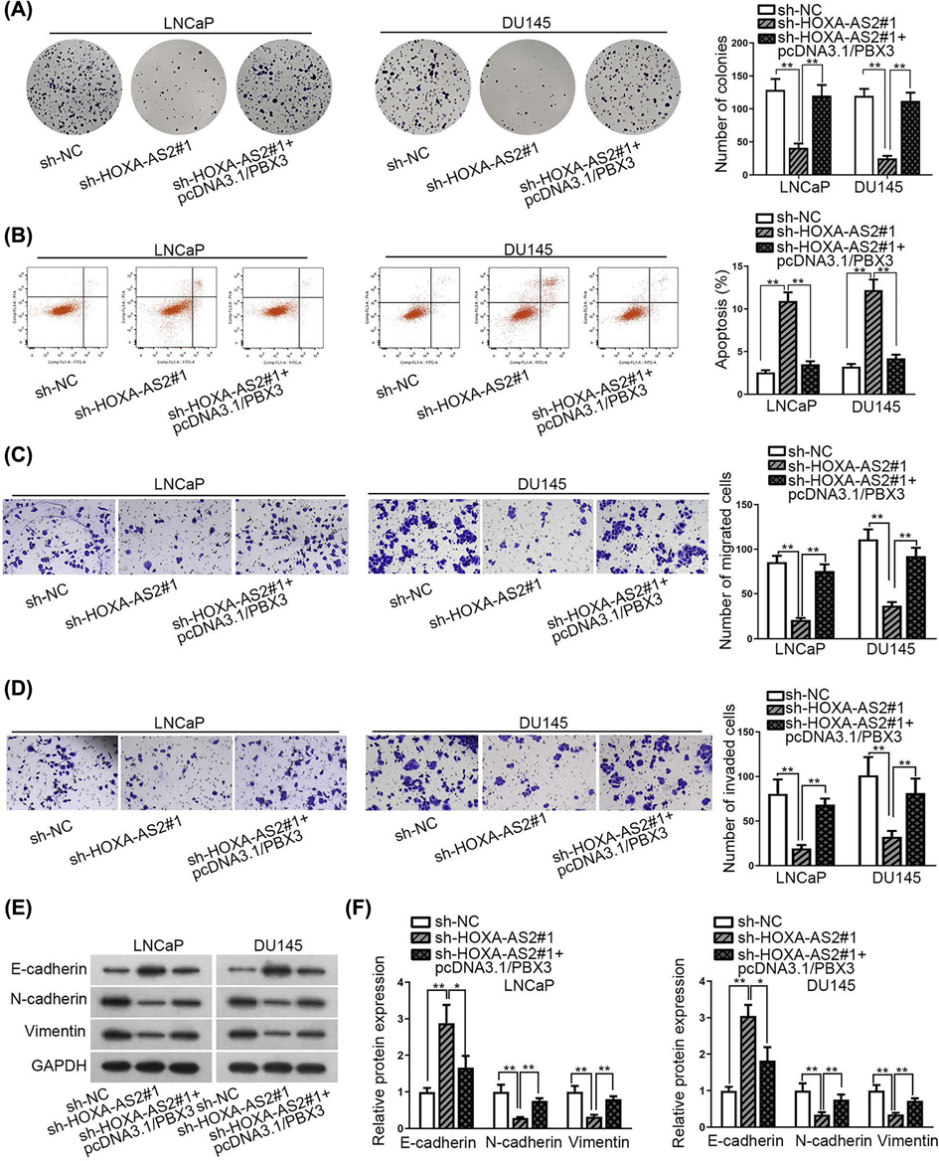
HOXA-AS2-miR-509-3p-PBX3 axis modulated the progression of PCa (**A,B**) Colony formation assay and flow cytometry were utilized to assess cell proliferation and cell apoptosis, respectively. (**C,D**) Transwell assay was implemented to test the migration and invasion of LNCaP and DU145 cells. (**E,F**) EMT process in prostate cells was evaluated via qRT-PCR and Western blot analyses. All results were shown as the mean ± SD. *P<0.05, **P<0.01.

Finally, rescue experiments were implemented to discover the functional system of HOXA-AS2-miR-509-3p-PBX3 axis in PCa. At first, we found that number of colonies reduced by depleting HOXA-AS2 was restored by the overexpression of PBX3 (Figure 5A). Meanwhile, increased cell apoptosis rate in HOXA-AS2-silenced cells was decreased by the overexpression of PBX3 (Figure 5B). Moreover, knockdown of HOXA-AS2 promoted the decline of cell migration and invasion, whereas overexpressing PBX3 restored this result (Figure 5C,D). Simultaneously, results of Western blot assay and qRT-PCR analysis revealed that EMT progression inhibited by silencing HOXA-AS2 was partially restored by the up-regulation of PBX3 (Figure 5E,F). These results displayed that HOXA-AS2-miR-509-3p-PBX3 axis played an oncogenic role in PCa.

## Discussion

It is increasingly obvious that lncRNAs were proofed to perform a crucial role in multiple human pathophysiologic progression, including tumorigenesis [25,26]. A few papers have uncovered that there were various lncRNA expressions in PCa [27–30]. HOXA-AS2 was initially determined in all-*trans* retinoic acid-treated NB4 promyelocytic leukemia cells. The molecular mechanism of HOXA-AS2 was depicted in multiple kinds of tumors [31]. Lian et al. revealed that HOXA-AS2-EZH2-LSD1 axis could exert cancer-promoting effect on pancreatic cancer cell growth [32]. It may provide a latent treatment for pancreatic cancer patients [32]. Gao et al. discovered that the depletion of reducing HOXA-AS2 repressed glioma cell actions and VM formation through targeting miR-373/EGFR axis [33]. Being consistent with previous findings, this study denoted that HOXA-AS2 expression was obviously high in PCa tissues. The patient survival rate with high-HOXA-AS2 expression was less than that with low-HOXA-AS2 expression. Functionally, cutting down HOXA-AS2 prevented PCa cells from proliferating and migrating. More importantly, silencing of HOXA-AS2 reversed the EMT process in PCa cells. These results suggested that HOXA-AS2 may play a tumor-promoting function in PCa.

It has reported that miR-509-3p served as a tumor inhibitor and played a significant role during the progression of several tumor categories, like breast cancer, acute lymphoblastic leukemia, renal cell carcinoma, as well as lung cancer etc [16–18]. For example, miR-509-3p could work as a cancer inhibitor in renal cancer [16]. MiR-509-3p expression was much lower in renal cancer cells than that in normal groups. In epithelial ovarian cancer, miR-509-3p could immediately target XIAP by its 3¢-UTR [34]. Here, we discovered that miR-509-3p expression was opposite to HOXA-AS2 in same samples. In addition, HOXA-AS2 could negatively modulate miR-509-3p expression through sponging with it.

It has been reported that PBX proteins were engaged in a number of tumors and played important parts in the progression of human cancer. Let-7c acted as a tumor inhibitor via depleting the expression level of PBX3 [35]. PBX3 facilitated cell proliferation and colony formation ability in gastric cancer [24]. Moreover, PBX3 expression was strongly related with lymph node invasion and poor prognosis in colorectal cancer [20]. These findings suggested that PBX3 played as an oncogenic role. Similarly, we also determined that PBX3 expression was much higher in PCa cells. MiR-509-3p could target PBX3 thus down-regulating PBX3. Furthermore, overexpressing PBX3 rescued the colony formation, migration and invasion impaired by HOXA-AS2 silence. So we could draw a conclusion that PBX3 played a cancer-promoting role as a target gene in PCa.

To sum up, lncRNA HOXA-AS2 indirectly regulate the expression of PBX3 through binding with miR-509-3p. The present study may provide some reference for new therapeutic strategy in PCa.

## Supplementary Material

Supplementary Figure S1

## Abbreviations

Ago2: argonaute2
ceRNA: competing endogenous RNA
EGFR: epidermal growth factor receptor
EMT: epithelial-to-mesenchymal transition
EZH2: enhancer of zeste 2 polycomb repressive complex 2 subunit
FBS: fetal bovine serum
FITC: fluorescein isothiocyanate
GAPDH: glyceraldehyde-3-phosphate dehydrogenase
HOXA: homeobox A cluster
HOXA-AS2: HOXA cluster antisense RNA 2
lncRNA: long non-coding RNA
LSD1: lysine-specific demethylase 1
miRNA: microRNA
mRNA: messenger RNA
NC: negative control
PBX3: pre-B-cell leukemia homeobox 3
PCa: prostate cancer
qRT-PCR: quantitative real-time polymerase chain reaction
RIP: RNA immunoprecipitation
VM: Vasculogenic Mimicry
XIAP: X-linked inhibitor of apoptosis
