# Supplementary material for: LncRNA HOXA-AS2 promotes the progression of prostate cancer via targeting miR-509-3p/PBX3 axis
Source: Biosci Rep. 2020 Aug 13;40(8):BSR20193287. doi: 10.1042/BSR20193287 (PMC7426630; doi:10.1042/BSR20193287)
Supplement: Supplementary Figure S1 [file BSR-2019-3287_supp.pdf]

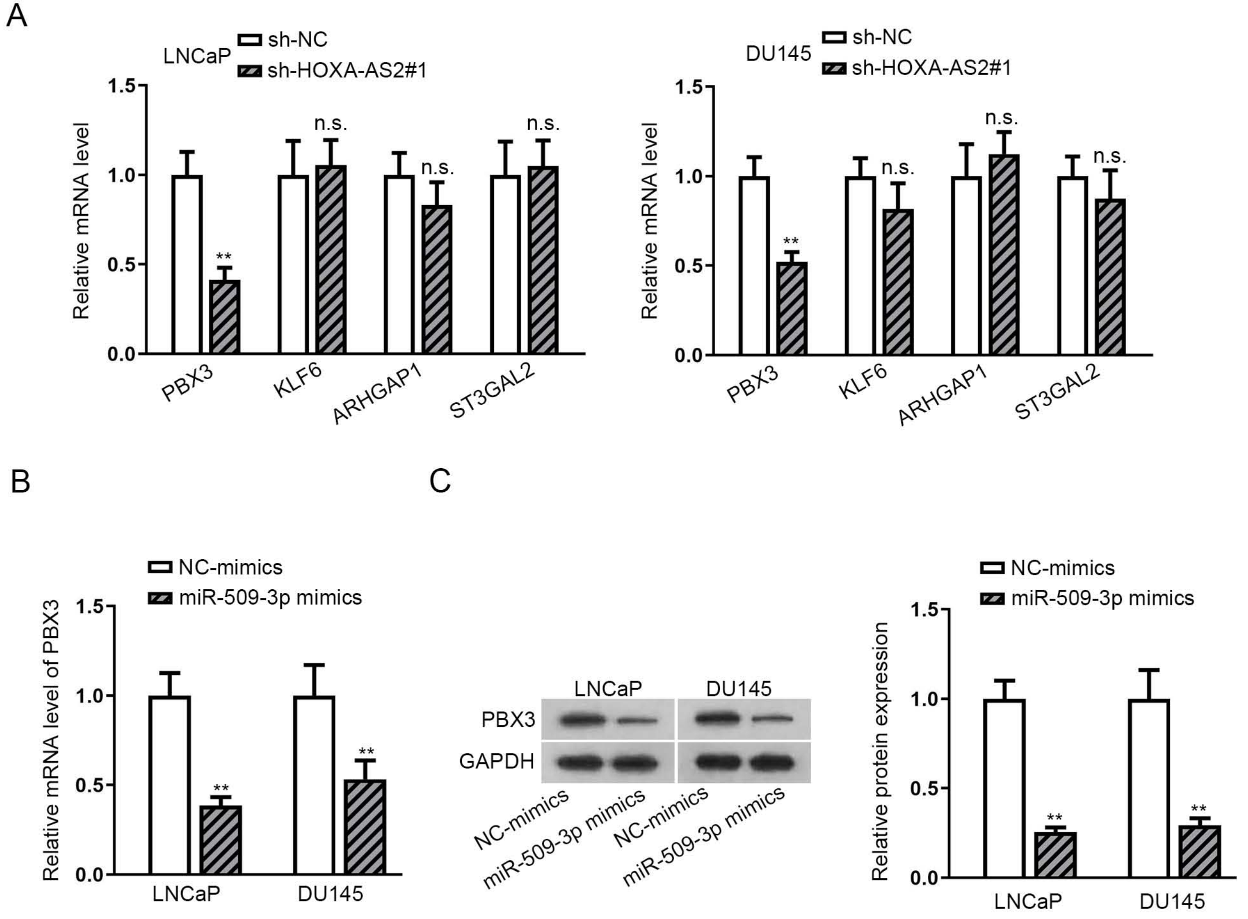
A
LNCaP
sh-NC
sh-HOXA-AS2#1
Relative mRNA level
1.5
1.0
0.5
0.0
n.s.
**
PBX3
KLF6
ARHGAP1
ST3GAL2
DU145
sh-NC
sh-HOXA-AS2#1
Relative mRNA level
B
NC-mimics
miR-509-3p mimics
Relative mRNA level of PBX3
LNCaP
DU145
C
LNCaP
DU145
PBX3
GAPDH
NC-mimics
miR-509-3p mimics
NC-mimics
miR-509-3p mimics
Relative protein expression

Figure S1. MiR-509-3p inhibited PBX3expression in prostate cancercells. A. The expressions of four candidate targets of miR-509-3p in cells transfected with sh-HOXA-AS2#1 or sh-NC. B. qRT-PCR data of PBX3 mRNA levelin LNCaP and DU145 cells transfected with miR-509-3p mimics orNC mimics. C. Western blot analysis of PBX3 protein in response to miR-509-3p overexpression.All results were shown as the mean ± SD. **P < 0.01, n.s.: no significance.
